# Spatial Variations and Determinants of Anemia among Under-five Children in Nepal, DHS (2006–2016)

**DOI:** 10.3390/ijerph19148664

**Published:** 2022-07-16

**Authors:** Shristi Sharma, Bipin Kumar Acharya, Qian Wu

**Affiliations:** 1 Health Science Center, Department of Epidemiology, School of Public Health, Xian Jiaotong University, Xi’an 710061, China; cristisrma592@stu.xjtu.edu.cn; 2 Institute of Fundamental Research and Studies, Kathmandu 44600, Nepal; acharyageog@gmail.com; 3 Nepal Geographical Society, Kathmandu 44600, Nepal; 4 Center for Geospatial Research and Development, Kathmandu 44600, Nepal

**Keywords:** anemia, Nepal Demographic Health Survey, under-5 children

## Abstract

Anemia among under-five children is the major health problem in Nepal. The lack of nutritional supplementation and lack of healthcare facilities are influential factors of anemia. Thus, the main objective of this study is to explore spatial variations and determinants of anemia among under-five children in Nepal. Nepal Demographic and Health Survey (NDHS) data from 2006 to 2016 were used in this study, which includes: household and individual-level data of 8555 under-five children, whose anemia was measured. In addition, a total of 260 (2006), 281 (2011), and 383 DHS clusters (2016) were taken in consideration for spatial analysis. The overall prevalence of anemia was 48.9%, 46.4%, and 52.2% in 2006, 2011, and 2016 respectively. The spatial analysis revealed a nonrandom spatial distribution, where statistically significant hotspots and coldspots were detected in different parts of the country. The results also identified mother’s age, mother’s educational level, socioeconomic status of household, number of under-5 children, household size, birth weight, underweight, stunting, diarrhea, and fever as associated factors of anemia among under-5 children. These findings may provide assistance to concerned health officials in adopting anemia-related programs and policies to address the anemia problems that plague Nepalese children under the age of five.

## 1. Introduction

Anemia occurs when the amount of red blood cells (and thus their oxygen-carrying capability) is insufficient to meet the body’s physiologic requirements [1]. Anemia inhibits blood oxygen circulation, which has negative consequences for maternal and child health, stunted child growth, decreased cognition, and reduced work productivity and money earning in adulthood [2]. It is a global public health problem that affects around 1.62 billion (24.8 percent) people globally, with 89 percent of cases happening in developing countries and the majority of cases occurring in children less than five-years-old and in reproductive-age women [3,4]. Children between 6 and 59 months (usually referred to as under-fives) with hemoglobin below the threshold of 11.0 (g/ld.) are considered to be anemic [5]. According to the WHO (World Health Organization), the global prevalence of anemia among children under the age of five was 39.8%, with the South Asia region having the second highest prevalence of 49.0% and Nepal having the fourth highest prevalence of 44.6 percent in South Asia [6]. Anemia in children under the age of five is a particular instance given its relevance in underlying a variety of morbidities and mortality within this population subset, albeit being important across the lifespan. These tendencies are alarming not just because they are incredibly treatable and preventive, but also because they hint at possible long-term ramifications for both individuals and society. Childhood anemia affects each person differently, impairing learning, morbidity, and mortality as well as motor and cognitive development. Strong evidence suggests that anemia negatively affects a nation’s socioeconomic well-being and productivity at the societal level [7,8].

Anemia is caused by multiple factors, including genetic abnormalities in hemoglobin genes, acute and chronic blood loss, insufficient dietary intake, viral infections, malaria, tuberculosis, and parasitic infections [9,10]. Iron deficiency anemia is the most common cause of anemia worldwide and results from the inadequate iron supply for erythropoiesis. Iron deficiency is particularly common during phases of fast body growth, such as in childhood and puberty [11]. Aside from these multiple clinical causes: structural and environmental variables, neighborhood, and household conditions, an individual’s health and nutritional level all influence anemia in children [12,13]. Unclean fuel use, poor toilet facilities, staying in a nonconcrete house, and exposure to smoking were important variables determining the prevalence of anemia in India [12]. In Ethiopia, age, parent’s education, birth order, wealth index, and household’s size were linked with anemia [14]. Regular anemia screening programs for children and population-based data on anemia are still lacking in Nepal. Anemia is frequently discovered when a child visits a health center for another reason [15]. In addition, limited attempts have been carried out to discover the determinants and trends of under-five anemia. Stunting, mother’s age, mother’s anemia status, child age, and other characteristics were shown to be linked with anemia in Nepalese children aged 5-years-old in a study conducted using DHS survey data in 2011, with nearly half of participants reported being anemic [16].

Similarly, understanding the regional variation of any disease is crucial for any country to prioritizes areas. Measures to provide public services by prioritizing areas, especially in developing nations, would benefit from a greater knowledge of the relationships between sociodemographic, economic, environmental, and health status variables. These relationships have a tendency to be quite unstable both within and between areas under varied situations. Regional administrative divisions may set different boundaries in various directions across sectors and locations [17]. In a study conducted in Nepal among reproductive-age women, the spatial analysis showed that statistically significant hotspots of anemia were in the southern Terai region (four districts in province 1, eight districts in province 2, one district in Bagmati province, two districts in province 5, and one district in Sudurpaschim province) [18]. However, no effort has been made to date in Nepal to discover regional variations of under-five anemia, despite the fact that understanding spatial variations and associated variables could be critical for a country’s development. Thus, the main objective of this study is to identify the regional variations and determinants of anemia in children, which will help to prioritize areas accordingly and plan and implement policy in the prioritized area to further reduce anemia.

## 2. Materials and Methods

### 2.1. Study Setting

Nepal is a small mountainous landlocked country situated in the southern slope of Himalaya. In 2015, after administrative changes, Nepal was divided into seven provinces, 77 districts, and 753 municipalities [19] (Supplement Figure S1). Municipalities are further divided into wards, which is lowest administrative unit in Nepal. Depending on the size of the population and economic activities, municipalities are classified as metropolitan, sub-metropolitan, municipality, and rural municipality in descending hierarchy.

### 2.2. Data Source

This study used Nepal Demographic Health Survey (NDHS) data for the years of 2006, 2011, and 2016. In brief, the NDHS is a cross-sectional nationally representative survey conducted in five years with collaboration between New ERA Nepal, Ministry of Health (MOH), Nepal, ICF International USA, and USAID [20]. SPSS data files (with individual rows containing household and individual-level information of under-five children) and shapefiles for the location of clusters visited (during the surveys) were specifically used for this study.

### 2.3. Sampling Method and Sample Size

Participants of this survey were selected using stratified two-stage cluster sampling in rural areas and three-stage cluster sampling in urban areas. In rural areas, wards were selected as primary sampling units (PSUs), and households were selected from the sample PSUs. In urban areas, wards were selected as PSUs, one enumeration area (EA) was selected from each PSU, and then households were selected from the sample EAs [21,22,23]. 

In this study, a total of 8885 (4692, 2088, and 2105 from 2006, 2011, and 2016, respectively) under-five children whose anemia level was determined were included. Likewise, a total of 260 DHS clusters (2006 and 2011) and 383 DHS clusters (2016) were taken in consideration for spatial analysis. 

### 2.4. Outcome Variable

The HemoCue instrument was used to determine anemia among under-five children. Based on the WHO hemoglobin level cut-off points, a hemoglobin level of 10.0–10.9 g/dL is defined as mild, 7.0–9.9 g/dL is moderate, and a level less than 7.0 g/dL is severe anemia. Therefore, a hemoglobin level of less than 11 g/dL of blood was considered anemic [24]. 

### 2.5. Explanatory Variables

Potential independent variables were selected based on previous studies [14,25,26,27,28] and information available on the NDHS survey. Selected variables were classified as individual-level factors and household factors. Sex, age, size at birth (birth weight less than 2.5 kgs is reported to be very small or smaller than average), stunting (height for age less than -2SD from the median of reference population), wasting (weight for height less than -2SD from the median of reference population), underweight (weight for age less than -2SD from the median of reference population), birth order, fever, diarrhea, cough, child twin status, birth intervals, mother’s and father’s educational level, age of mother, marital status, mothers’ working status, religion, and mother’s media exposure were selected individual factors. 

Likewise, place of residence (urban, rural), source of drinking water, type of toilet facility, type of cooking fuel, number of under-five children in the family, household size, and wealth-index were selected. The wealth index is a composite measure of a household’s cumulative living standard. The wealth index is calculated using easy-to-collect data on a household’s ownership of selected assets, such as televisions and bicycles; materials used for housing construction; types of water access and sanitation facilities [29].

### 2.6. Data Analysis

The prevalence of anemia and children was calculated using reported sample weights. Frequency and percentage were used to describe the sample’s characteristics (household, individual, and contextual). First, we identified factors that were associated with anemia, using the chi-square test. Thereafter, univariate logistic regression analysis was performed to identify the effects of these factors resulting in anemia by computing the odds ratio. A *p*-value less than 0.05 was considered statistically significant.

For the spatial analysis, anemia data were linked with cluster locations using the common cluster id column of both DHS data and DHS location data. We then computed the anemia prevalence for each DHS cluster and each survey year. The cluster-level prevalence was visualized using the Arc GIS.

The Global Moran’s I [30] and Getis-Ord Gi [31] were used to assess overall spatial patterns and the local-level spatial variation and heterogeneity of anemia among under-5 children, respectively. The Global Moran’s I is a widely used indicator of spatial autocorrelation and independence. Its value ranges from −1 to 1, where 1 indicates a perfect positive correlation, 0 implies perfect spatial randomness, and −1 suggests a perfect negative spatial autocorrelation. The significance was tested at 90, 95 and 99 % confidence using the z test. 

The local-level variations and heterogeneity were assessed using hotspot analysis based on the Getis-Ord Gi* statistic. Unlike the Global Moran’s I, Getis-Ord Gi* statistics provides z scores and associated p values of each of the observations. Therefore, it can map statistically significant hotspots or coldspots deepening upon positive or negative z values, respectively, over the study region. The z values near to zero are considered insignificant. 

## 3. Results

### 3.1. Sociodemographics Chacterictics

Table 1 illustrates the weighted frequency and percentages of sociodemographic characteristics of mother–child pairs included in our study. Among 8885 mothers, 47.9% were 25–34 years and 39.3% were 15–24 years. More than half of mothers (52.0%) had no education, whereas 27.9% of fathers did not have any education. A percentage of 32.8% of participants were from the central development region, which is 79.0% of total women living in a rural area. The majority of them (84.9%) followed the Hindu religion and 31.3% were of Brahmin/Chettri ethnicity. The majority of them have 1 or 2 children (85.5) but most of them live in a family with 5 members or more (72.6%). A percentage of 24.7% of HHs were in the poorest range followed by poorer (21.5%) and middle (21.4%).

### 3.2. Under-Five Children Charcteristics

Among the 8885 under-5 children, the majority (67.6%) were above 24 months, followed by 12–13 months (22.0%). Fifty-one percent of children were male. In addition, 62.4% of them had a normal birth size but 17.8% of them had a less than average size. A percentage of 32.3% of them were first birth and 75.8% were last birth. In anthropometric measurements, 35.4%, 11.4%, and 47.3% of them were underweight, wasted, and stunted, respectively. Table 2 also illustrates that 19.2%, 11.4%, and 20.0% of them reported fever and diarrhea (Table 2).

### 3.3. Prevalence of Anemia among Under-5 Children in Nepal

**Figure 1:**
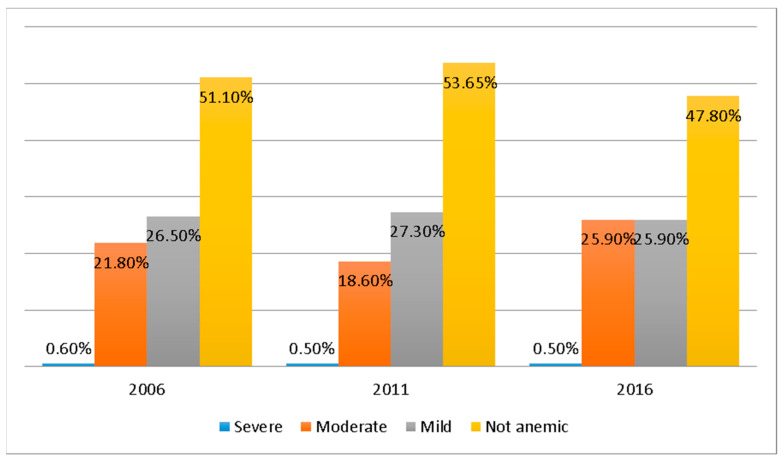
Prevalence of anemia among under-five children in Nepal (NDHS 2006–2016).

The overall prevalence of anemia was 48.9% in 2006, 46.4% in 2011, and 52.2% in 2016. Among all the children in our study, 0.6% were severe, 22.0% were moderate, 26.5% were mild, and 50.9% were not anemic (Figure 1).

### 3.4. Spatial Variation of Anemia among Under-5 Children in Nepal

The spatial analysis based on the Moran’s I index revealed a nonrandom spatial distribution of anemia prevalence over the study region (Table 3) in all the DHS surveys. However, the degree of spatial dependence varies over the survey year.

**Figure 2:**
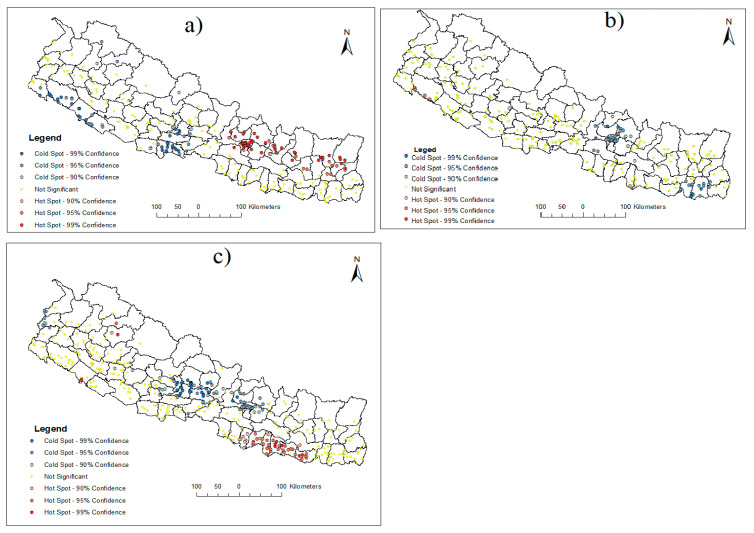
Spatial distribution of hotspots and coldspots of anemia among under-five children in Nepal, 2006 (**a**), 2011 (**b**), and 2016 (**c**). (Note: “Anemia was measured using a hemo-cue device which has gone through technical changes over the years to improve measurement precision, thus limiting the data comparability over the years”).

The Getis-Ord-G* hotspot analysis revealed both hotspots and coldspots in 2006 NDHS data. Out of 260 DHS clusters, 62 were identified as hotspot clusters while 19 were identified as coldspots. The hotspots were observed around Kathmandu Valley, as well as eastern mountain and hill districts, while coldspots were found distributed in the mid- and far-western Terai region of Nepal (Figure 2A). In 2011, only 8 DHS clusters distributed in the central and western Terai were detected as significant hotspots, while there were 56 coldspot DHS clusters. The coldspots were around Kathmandu valley and eastern Terai, especially in Morang district (Figure 2B). Similarly, both hotspots and coldspots were found among 383 DHS clusters in 2016. The numbers of DHS clusters identified as hotspots were 50, while this number was 69 for the coldspots. In 2016, hotspot clusters were mainly distributed in many districts of central Terai, while the spatial patterns of coldspots were around Kathmandu and Kaski (Figure 2C).

### 3.5. Determinants of Anemia among Under-5 Children

Univariate logistics analysis was used to identify potential determinants for under-5 anemia (Table 4). Mothers with an age less than 24 have a high chance of having a child with anemia (OR = 1.731, 1.132–2.648). In addition, the odds of having a child with anemia increases if the level of education of the mother and father decreases (OR > 1). Household wealth index was significantly associated with anemia: the poorest wealth index (OR = 1.333, CI = 1.160–1.533) has a high chance of having anemia. Similarly, a household with less than 4 members (OR = 0.767, CI = 0.685–0.860) and 2 under-five children (OR = 0.774, CI = 0.689–0.869) has a lower chance of having anemia than with HHs of more than 8 members and 2 children.

Children who were 12 to 23 months (OR = 3.462, CI = 3.107–3.857) and 6 to 11 months (OR = 5.327, CI = 4.538–6.253) have higher chances of having anemia than those more than 24 months. Children who have large sizes have comparatively fewer chances of having anemia than those with small birth sizes (OR = 0.782, CI = 0.684–0.895). In addition, chances of anemia decrease if a child does not have a twin (OR = 0.588, CI = 0.383–0.934). In addition, children who are stunted (OR = 1.203, CI = 1.107–1.308), wasted (OR = 1.533, CI = 1.342–1.752), and under-weight (OR = 1.358, CI = 1.245–1.482) have higher chances of anemia than with those who are not, and those who did not have fever (OR = 0.807, CI = 0.726–0.869), cough (OR = 0.794, CI = 0.715–0.875), and diarrhea (OR = 0.686, CI = 0.601–0.783) had fewer chances of having anemia. In comparison to 2006, anemia chances increased in 2016 significantly (OR = 1.157, CI = 1.044–1.283).

## 4. Discussion

Anemia is one of the serious public health problems among under-five children in Nepal. This study analyzed the spatial variation of under-5 anemia using the NDHS data for the years of 2006, 2011, and 2016. The results revealed that nearly 50 percent of children under 5 years of age were anemic. The spatial distribution of the prevalence of anemia of less than 5 years is nonrandom and statistically significant hot/coldspots were observed in different parts of the country over the DHS years. A number of socioeconomic factors including mother’s age, mother’s educational level, socioeconomic status of household, number of under-5 children in household, household size, birth weight, stunting, underweight, stunting, diarrhea, and fever were found significantly associated with anemia. 

As per the latest DHS survey data, the prevalence of under-five anemia was 52.6% in Nepal. The prevalence of under-five anemia, although lower in some other Southeast Asian countries such as India (58.5% in 2016), Myanmar (57.8% in 2016), and Cambodia (55.5% in 2014) [32,33], is still alarming because, as our study suggests, considerable increases in the prevalence of anemia in 2016 after a decline in numbers had been reported between 2006 and 2011. This suggests that “National Anemia Control strategies” drafted in 2009 and the “Nepal Health Sector programme 2010–2015” with the aim of reducing under-five anemia to forty-two percent have not been achieved yet [34,35]. The reason for the rise in prevalence in 2016 is difficult to pinpoint, although one of the suggested causes could be sample bias, which is unavoidable with DHS surveys [36], also because the sampling unit in Nepal was changed in 2016 due to a restructure of the administrative division in 2015. Another likely cause could be the 2015 earthquake in Nepal: during this time, essential elements such as food security and food shortages can lead to serious protein energy malnutrition and other deficiencies, which can exacerbate anemia’s burden [37,38]. However, this definitely opens the area to explore and identify the underlying cause for this disparity in the near future as a similar trend was also observed in Ethiopia (increased in 2016 after decline in 2011) [14].

Spatial analysis revealed the nonrandom distribution of anemia prevalence in all the DHS surveys. The spatial dependency in 2006 and 2016 is more pronounced than that in the year of 2011. In 2016, the hotspots were observed in different districts of central east Terai, while coldspots were observed around Kathmandu and Kaski regions. Possible reasons include a lack of safe and appropriate drinking water in the Terai region, which increases the probability of malaria and hookworm infection, as well as a lack of variety in the Terai diet, which increases the risk of anemia [18,39,40,41]. In addition, Kaski and Pokhara ranked last and fourth last among all districts in Nepal in poverty rate as per the small areas poverty estimation of 2016 [42]. Thus, this could be the reason for coldspots around these areas. No hotspots were observed at the neighborhood level in the 2011 survey, but coldspots were observed around Kathmandu and Sunsari areas. Susari also had a lower poverty rate than other districts [42]. The emergence of these coldspots can be linked with the 2016 survey and can be interpreted with wealth and education. Contrary to the recent DHS survey, the spatial distribution of hotspots around Kathmandu in 2006 is unrealistic and hard to explain. However, hotspots in the eastern mountain region in 2006 can be explained by poor healthcare delivery in these areas [18], which have improved in recent decades [43].

The age of the mother was revealed to be one of the strongest factors of anemia. Young moms (15–24) have a higher risk of anemia in their children. This is in line with the findings of an Ethiopian study [14]. This could be due to the fact that early pregnancy increases the risk of low-birth-weight kids, which is connected to inadequate nutrition and anemia [25]. In addition, mothers over 45 had a higher risk of producing anemic children. Increasing maternal age is also linked to a reduced ability for fetal growth, which can result in anemia in offspring [25]. In a similar way to India, we discovered a link between the degree of education of the mother and childhood anemia [12,26]. This might be supported by a study that concluded that the influence of the mother’s education on the adopted sample is similar to that on the own birth sample [27].

The socioeconomic status of the households showed an association with anemia. The chances of having anemia increase as a household status changes from richest to poorest. This could be explained by studies that show that wealth inequality is strongly associated with chronic childhood undernutrition [44,45]. Likewise, it is a well-known fact that an increase in the number of children might lead to a risk of communicable disease transmission, and competition for food and, consequently, nutritional deficiencies [46]. Our study also shows that the prevalence of anemia increases with the increase in under-five children in the household. 

Unlike India, Ethiopia, Rwanda, and Bangladesh did not show any significant association between HHs and anemia [12,14,46,47]; we reported that HH size was strongly associated with anemia, where the risk increases as the size increases. However, surprisingly, in a study conducted in north-western Uganda, the risk of anemia decreased with increasing household [48]. This inconsistent result might hint that there might be other external factors affecting the relation between HHs and anemia, which might be needed for further exploration. 

Although absorbed iron requirements increase with age [49], our study shows that the prevalence of anemia is lower for children above 24 months than younger ones. This could be because children above the age of one year eat a variety of foods high in iron, such as meats, poultry, fish, and cereals, which could be used as a supplement to meet the increased anemia requirements [50,51].

Birth weight, stunting, wasting, and underweight are strong indicators of malnutrition, and all of these variables are strongly related to anemia in our study (*p* < 0.01). These are the same factors that have been found to be risk factors in numerous studies [14,15,31,46]. Nutritional inadequacies may also impair immunity, which, in turn, can have associations with low concentrations of hemoglobin (anemia) [52]. 

Having diarrhea and fever is also associated with anemia in this study. Present diarrhea, current fever, and a history of diarrhea in the past seven days were all linked to an elevated risk of anemia in an Indonesian study [53]. Although the origin of fever is unknown, a fall in hemoglobin content may be connected to a probable illness that compromises immunity [54]. Moreover, in diarrhea, loss of nutrition can lead to low iron concentration, which ultimately results in anemia [55].

The potential strength of our study is the use of all available data (NDHS 2006 to 2016); this enables us to have a large sample size. In addition, geospatial analysis allows us to gain a better insight to locate the high- and low-hotspot areas of anemia across the country. Likewise, this study fills gaps in the knowledge relating to social determinants of childhood anemia in Nepal. Despite its strength, these findings should be interpreted considering its limitations. Some of the independent variables such as birth size, weight, fever, and diarrhea are taken subjectively, which might lead to recall bias. In addition, as this is a self-report survey and uses the census method, census bias and information bias cannot be inevitable. Similarly, our study uses a HemoCue device for measurement of anemia, which has had some technical changes over the years for improving the precision of measurement, thus limiting accurate comparability over the years. 

## 5. Conclusions

This study shows the prevalence of anemia among children in Nepal in different DHS survey years from 2006 to 2016 where the national average prevalence rate is around 50 percent (48.9% in 2006 and 46.4% in 2011 and 52.2% in 2016). Spatial analysis reveals the nonrandom distribution with statistically significant hotspots and coldspots in different parts of the county. Several factors including mother’s age, mother’s educational level, socioeconomic status of household, number of under-5 children, household size, birth weight, underweight, stunting, diarrhea, and fever were found associated with anemia. Findings of this study could be useful in the formulation strategies related with childhood anemia in Nepal. The government intervention strategies should be spatially explicit and more resources should be allocated in the hotspots explored in this study as present strategies seem to be inadequate, to lower the prevalence and underlining cause of anemia. Further, the disparity of results, seen across the different time periods in different regions in this study, suggest the need of further studies to be conducted in this area.

## Figures and Tables

**Table 1:** Sociodemographic characteristics of respondents/households, *n* = 8885.

Characteristics	Frequency	Percentage
**Mother’s Age**		
15–24	3412	39.3
25–34	4156	47.9
35–44	1016	11.7
Above 45	91	1.1
**Religion**		
Hindu	7367	84.9
Buddhist	589	6.8
Muslim	478	5.5
Others	239	2.8
**Education level of mother**		
No	4513	52.0
Primary	1612	18.6
Secondary	2078	24.0
Higher	472	5.4
**Education level of father**		
No	2422	27.9
Primary	2663	30.7
Secondary	1691	19.5
Higher	1385	16.0
Don’t know	514	5.9
**Type of residence**		
Urban	1824	21.0
Rural	6850	79.0
**Ethnicity**		
Brahmin/Chettri	2715	31.3
Janajati	1674	19.2
Terai others	1065	12.3
Dalit	1047	12.1
Muslim	349	4.0
Newar	342	3.9
Others	1483	17.2
**Wealth Index**		
Poorest	2142	24.7
Poorer	1869	21.5
Middle	1854	21.4
Richer	1598	18.4
Richest	1212	14.0
**No. of under-five children**		
1 to 2	7417	85.5
3 and above	1258	14.5
**No. of HH size**		
2 to 4	2372	27.3
5 to 7	3968	45.7
8 and above	2332	26.9
**Development Region**		
Eastern	1944	22.4
Central	2848	32.8
Western	2019	23.3
Mid-Western	1014	11.7
Far Western	850	9.8

**Table 2:** Characteristics of under-5 children in Nepal, *n* = 8885.

Characteristics	Frequency	Percentage
**Age in months**		
6–11	900	10.4
12–23	1911	22.0
24–59	5864	67.6
**Sex**		
Male	4443	51.1
Female	4242	48.9
**Child is twin**		
Yes	75	0.9
No	8600	99.1
**Birth Size**		
Very small	407	4.7
Small	1136	13.1
Average	5416	62.4
Larger and very large	1708	19.7
Don’t know	8	0.1
**Preceding birth interval**		
24 months and less	1448	16.7
More than 24 months	4404	50.8
First birth	2823	32.5
**Succeeding birth interval**		
Last birth	880	75.8
24 months and less	1217	10.1
More than 24 months	6578	45.0
**Underweight**		
No	5559	64.1
Yes	3075	35.4
**Wasting**		
No	7637	88.0
Yes	992	11.4
**Stunting**		
No	4525	52.2
Yes	4104	47.3
**Fever Last 2 weeks**		
No	7012	80.8
Yes	1661	19.2
**Diarrhea last 2 week**		
No	7682	88.6
Yes	987	11.4
**Cough last 2 week**		
No	6935	79.9
Yes	1738	20.0

**Table 3:** Results of the spatial autocorrelation analysis.

	2005	2011	2016
Moran’s Index	0.170688	0.018449	0.157314
Expected Index	−0.003861	−0.003472	−0.002618
*p*	0.0000	0.331	00000
Z score	5.18	0.7405	5.4444

**Table 4:** Unadjusted association between predictor variables and anemia.

Characteristics	Odd Ratio	95% CI	*p* Value
**Mother’s age**			
15–24	1.731	1.132–2.648	<0.01
25–34	0.723	1.384–0.906	
35–44	0.299	1.261–0.814	
45 and above	1	1	
**Mother’s Education**			
No education	1.922	1.579–2.339	<0.01
Primary	1.510	1.222–1.866	
Secondary	1.575	1.281–1.936	
Higher	1	1	
**Father’s education**			
No education	1.370	1.200–1.565	<0.01
Primary	1.238	1.087–1410	
Secondary	1.086	0.942–1.253	
Higher	1	1	
**Wealth index**			<0.001
Poorest	1.333	1.160–1.533	
Poorer	1.532	1.323–1.774	
Middle	1.725	1.484–2.005	
Richer	1.481	1.272–1.726	
Richest	1	1	
**Household size**			<0.01
2 to 4	0.767	0.685–0.860	
5 to 7	0.850	0.768–0.940	
8 and above	1	1	
**No. of under-5 children**			<0.01
1 to 2	0.774	0.689–0.869	
3 and more	1	1	
**Age of child (in a month)**			
6 to 11	5.327	4.538–6.253	<0.001
12 to 23	3.462	3.107–3.857	
24 to 59	1	1	
**Child is twin**			
Yes	1	1	0.022
No	0.588	0.383–0.934	
**Birth size**			
Large than average	0.782	0.684–0.895	<0.01
Average	0.542	0.866–1.0079	
Small than average	1	1	
**Stunting**			
Yes	1.203	1.107–1.308	<0.01
No	1	1	
**Underweight**			
Yes	1.358	1.245–1.482	<0.01
No	1	1	
**Wasting**			
Yes	1.533	1.342–1.752	<0.01
No	1	1	
**Had fever**			
Yes	1	1	<0.01
No	0.807	0.726–0.869	
**Had diarrhea**			
Yes	1	1	<0.01
No	0.686	0.601–0.783	
**Had Cough**			
Yes	1	1	<0.01
No	0.794	0.715–0.875	
**Year of survey**			0.001
2006	1	1	
2011	0.922	0.832–1.023	
2016	1.157	1.044–1.283	
**Preceding birth interval**			
First birth	1	1	0.02
24 months and less	1.213	1.049–1.266	
More than 24 month	1.152	1.070–1.3751	
**Succeeding birth interval**			
24 and less	0482	0.426–0.546	<0.01
More than 24	0.562	0.488–0.648	
Last birth	1	1	

## Data Availability

https://www.dhsprogram.com/Data/ (accessed on 21 December 2020).

## Supplementary Materials

The following supporting information can be downloaded at: https://www.mdpi.com/article/10.3390/ijerph19148664/s1, Figure S1: Landmark of provinces and districts of Nepal.
