# Supplementary figures and images for: Spatial Variations and Determinants of Anemia among Under-five Children in Nepal, DHS (2006–2016)
Source: Int J Environ Res Public Health. 2022 Jul 16;19(14):8664. doi: 10.3390/ijerph19148664 (PMC9323660; doi:10.3390/ijerph19148664)

## Supplementary Materials

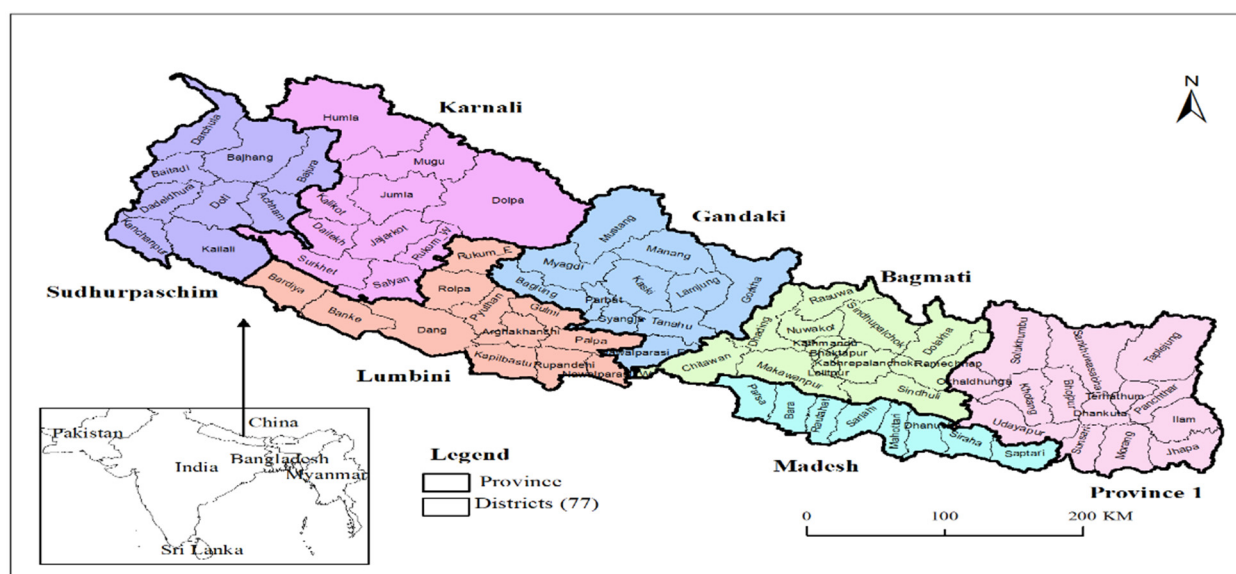

**Figure S1.** Landmark of provinces and districts of Nepal.

Supplement: Supplementary file 1 [file ijerph-19-08664-s001.zip › ijerph-1694293-SI.pdf]
